# Drimane Sesquiterpenoids and Isochromone Derivative from the Endophytic Fungus *Pestalotiopsis* sp. M-23

**DOI:** 10.1007/s13659-016-0094-6

**Published:** 2016-04-02

**Authors:** Ce Kuang, Shu-Xi Jing, Yan Liu, Shi-Hong Luo, Sheng-Hong Li

**Affiliations:** 1 State Key Laboratory of Phytochemistry and Plant Resources in West China, Kunming Institute of Botany, Chinese Academy of Sciences (CAS), Kunming, 650201 People’s Republic of China; 2 University of Chinese Academy of Sciences, Beijing, 100049 People’s Republic of China

**Keywords:** Endophytic fungi, *Pestalotiopsis* sp. M-23, *Leucosceptrum canum*, Drimane sesquiterpenoids, Antibacterial activity

## Abstract

**Abstract:**

Three new drimane sesquiterpenoids (**1**–**3**) together with the known 2*α*-hydroxyisodrimeninol (**4**), and a new isochromone derivative (**5**), were obtained from the solid cultures of fungal strain *Pestalotiopsis* sp. M-23, an endophytic fungus isolated from the leaves of *Leucosceptrum canum* (Labiatae). Their structures were determined by comprehensive 1D and 2D NMR, and MS analyses. The metabolites were evaluated for their antibacterial activities, and compound **3** showed weak inhibitory activity against *Bacillus subtilis*.

**Graphical Abstract:**

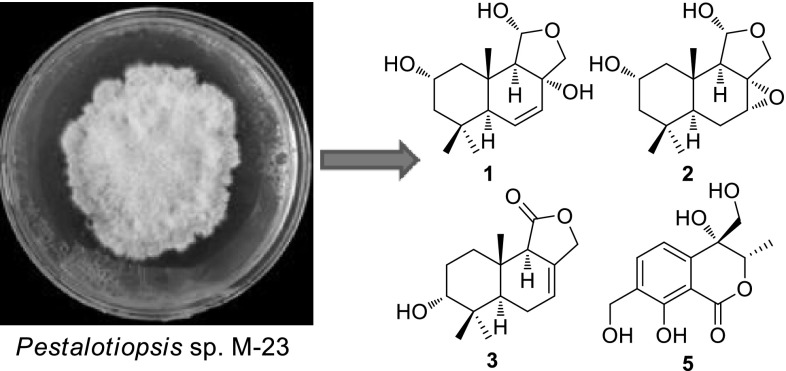

**Electronic supplementary material:**

The online version of this article (doi:10.1007/s13659-016-0094-6) contains supplementary material, which is available to authorized users.

## Introduction

Fungal endophytes are ubiquitous fungi that inhabit healthy plant tissues without causing apparent symptoms of disease, and their colonizations have been found in almost all kinds of plants, from algae to vascular plants [1]. Endophytic fungi have gained prominence as important sources of a variety of new biologically active natural products [2]. For example, the famous anticancer drug paclitaxel can be produced by the endophytic fungus *Taxomyces andreance* from Pacific yew [3]. An endophytic fungus *Thielavia subthermophila* [4] from the medicinal herb *Hypericum perforatum* has been reported to produce the antidepressant napthodianthrone derivative hypericin [5].

The fungal genus *Pestalotiopsis*, representing one of the largest biomasses of any plant-associated endophytic fungus in the world, produces a large variety of secondary metabolites, including terpenoids, alkaloids, chromone derivatives and phenolics [6], with cytotoxic [7], antibacterial and antifungal activities [8, 9]. In this study, an endophytic fungal strain, *Pestalotiopsis* sp. M-23, was isolated from the leaves of *Leucosceptrum canum*, a woody Labiatae (=Lamiaceae) plant with unique dark-brown colored nectar that has been proved to be caused by a novel bird attractant proline-benzoquinone, and with a unique class of sesterterpenoids named leucosceptroids that have been shown to function as defense against attack by insect herbivores and pathogens [10–14]. In this study, a phytochemical investigation on the solid culture of the endophytic fungus *Pestalotiopsis* sp. M-23 was carried out, which led to the isolation and identification of five compounds (**1**–**5**), including three new drimane sesquiterpenoids (**1**–**3**) and a new isochromone derivative (**5**). In addition, the antibacterial activities of these compounds were also evaluated.

## Results and Discussion

More than one hundred endophytic fungal strains were isolated from the leaves of *L. canum*. Among them, M-23 was identified as *Pestalotiopsis* sp. using molecular biological techniques [15]. To carry out phytochemical investigation, the mycelia and solid culture media of *Pestalotiopsis* sp. M-23 were extracted with acetone, and the crude extract was fractionated by column chromatography on silica gel. Further purification was performed by repeated normal-phase, Sephadex LH-20, ODS column chromatographies, and reverse-phase semi-preparative HPLC to yield five compounds (**1**–**5**).

**Fig. 1:**
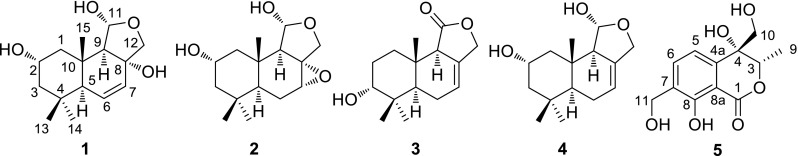
Chemical structures of compounds **1**–**5**

**Fig. 2:**
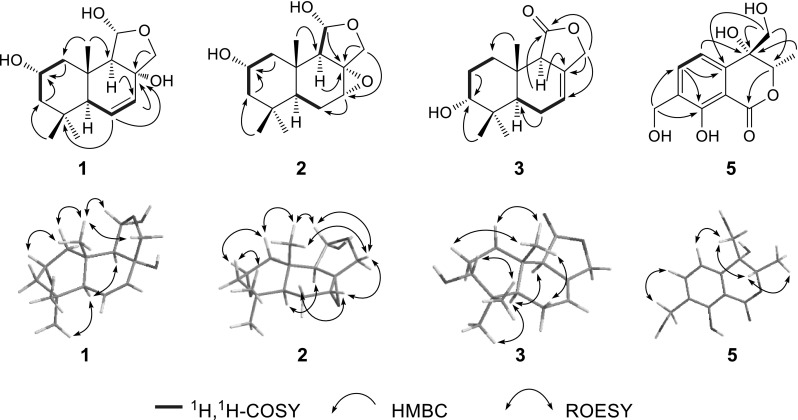
Major correlations in 2D NMR spectra of compounds **1**–**3** and **5**

Compound **1** was isolated as colorless oil. Its molecular formula was established as C_15_H_24_O_4_ by its HR-ESI-MS (*m/z* 291.1570, [M + Na]^+^) and ^13^C NMR spectroscopic data. The IR spectrum showed typical absorptions at 1630 and 3425 cm^−1^ for double bond and hydroxyl groups, respectively. In the ^1^H NMR spectrum (Table 1), three tertiary methyl resonances at *δ*_H_ 1.01, 0.94, and 0.88 (each 3H, s) were clearly shown. A pair of AB doublets was observed at *δ*_H_ 4.08 (d, *J* = 9.4 Hz) and 3.70 (d, *J* = 9.4 Hz), indicating the presence of an oxygenated methylene group. An AMX system was observed at *δ*_H_ 5.88 (dd, *J* = 9.8, 1.7 Hz), 5.78 (dd, *J* = 9.8, 2.8 Hz) and 1.93 (dd, *J* = 2.8, 1.7 Hz), implying the presence of an *endo*-double bond. The ^13^C NMR spectrum (Table 1) showed the presence of 15 carbon resonances which were classified using DEPT experiments into three methyls (*δ*_C_ at 33.4, 23.0, and 15.8), three methylenes (including one oxygenated methylene at *δ*_C_ 80.9), six methines (including a double bond group at *δ*_C_ 130.4 and 129.4; an oxymethine *δ*_C_ 64.8; and a hemiacetal methine at *δ*_C_ 101.7), and three quaternary carbons (including an oxygen-bearing carbon at *δ*_C_ 79.0). These data suggested that **1** was a drimane sesquiterpenoid [16, 17]. Comparing the NMR spectra (Table 1) of **1** with those of 2*α*-hydroxyisodrimeninol (**4**) [17, 18], a drimane sesquiterpenoid also isolated from this fungus, revealed that the two compounds were similar. Compound **1** showed the typical hemiacetal and oxymethine resonances as those in **4**. The major difference between these two compounds was that the tri-substituted double bond in **4** was replaced by a di-substituted double bond located between C-6 and C-7 in **1**, which was confirmed by the ^1^H-^1^H COSY correlations of H-6 (*δ*_H_ 5.88) with H-5 (*δ*_H_ 1.93) and H-7 (*δ*_H_ 5.78). An oxygenated quaternary carbon was found to occur at C-8 (*δ*_C_ 79.0) in **1** due to the HMBC correlations from H-7 (*δ*_H_ 5.78), H-9 (*δ*_H_ 2.07), and H_2_-12 (*δ*_H_ 3.70 and 4.08) to C-8 (Fig. 2). The relative configuration of **1** was deduced from the results of ROESY experiment and comparison with the data in literature [16, 17]. The ROESY correlations of Me-15 (*δ*_H_ 0.88) with H-2 (*δ*_H_ 3.91), H-11 (*δ*_H_ 5.37) and H_a_-12 (*δ*_H_ 3.70) revealed that these protons were *β*-oriented, and the ROESY correlation between H-5 (*δ*_H_ 1.93) and H-9 (*δ*_H_ 2.07) suggested their *α*-oriented configuration (Fig. 2). Consequently, the structure of **1** was determined as 2*α*,8*α*-dihydroxy-6,7-en-isodrimeninol (Fig. 1).

**Table 1:** ^1^H and ^13^C NMR data of **1**–**4** [*δ* in ppm; *J* in Hz]

Position	**1** ^a^		**2** ^a^		**3** ^b^		**4** ^b^	
	*δ*_H_^c^	*δ*_C_^d^	*δ*_H_^c^	*δ*_C_^d^	*δ*_H_^c^	*δ*_C_^e^	*δ*_H_^c^	*δ*_C_^e^
1*α*	1.13 m	48.2 t	1.03 m	49.8 t	1.91 m	31.3 t	1.17 m	49.9 t
1*β*	2.08 m		2.02 m		2.09 m		2.13 overlap	
2*α*	3.91 m	64.8 d	3.74 m	64.7 d	1.57 m	25.8 t	3.84 m	64.0 d
2*β*					1.94 m			
3*α*	1.17 m	51.0 t	1.12 m	51.9 t		75.5 d	1.17 m	52.4 t
3*β*	1.80 m		1.71 overlap		3.44 br s		1.76 m	
4	–	34.6 s	–	35.1 s	–	37.9 s	–	35.0 s
5	1.93 dd (2.8, 1.7)	52.7 d	0.93 overlap	44.9 d	1.89 m	43.6 d	1.28 m	50.2 d
6	5.88 dd (9.8, 1.7)	130.4 d	1.71 overlap	23.7 d	2.07 m	23.5 t	1.91 m	24.2 t
			2.05 m		2.11 m		2.13 overlap	
7	5.78 dd (9.8, 2.8)	129.4 d	3.38 br s	59.6 d	5.79 br s	121.3 d	5.51 br s	116.8 d
8	–	79.0 s	–	65.2 s	–	131.6 s	–	138.4 s
9	2.07 br s	70.2 d	1.70 d (1.9)	62.3 d	2.94 s	54.0 d	2.23 d (3.0)	62.5 d
10	–	40.9 s	–	36.0 s	–	34.5 s	–	35.6 s
11	5.37 br s	101.7 d	5.32 d (1.9)	100.1 d	–	175.5 s	5.22 d (3.0)	99.5 d
12a	3.70 d (9.4)	80.9 t	3.75 d (10.0)	68.0 t	4.63 d (11.7)	70.2 t	4.06 d (11.3)	68.5 t
12b	4.08 d (9.4)		4.00 d (10.0)		4.70 d (11.7)		4.35 d (11.3)	
13	0.94 s (3H)	23.0 q	0.97 s (3H)	23.4 q	0.93 s (3H)	22.1 q	0.91 s (3H)	22.7 q
14	1.01 s (3H)	33.4 q	0.92 s (3H)	33.3 q	0.96 s (3H)	28.7 q	0.94 s (3H)	33.5 q
15	0.88 s (3H)	15.8 q	0.92 s (3H)	16.2 q	0.86 s (3H)	14.3 q	0.85 s (3H)	15.2 q
3-OH					3.52 br s			

^a^Recorded in CD_3_OD

^b^Recorded in acetone-*d*_6_

^c^Recorded at 400 MHz

^d^Recorded at 150 MHz

^e^Recorded at 100 MHz

Compound **2** was obtained as colorless oil. It gave a molecular formula of C_15_H_24_O_4_ according to ^13^C NMR spectroscopic and HR-EI-MS data (*m/z* 268.1677 [M]^+^), with 4° of unsaturation. The ^1^H and ^13^C NMR spectroscopic data (Table 1) were similar to those of **4**, suggesting that **2** was also a drimane sesquiterpenoid with a hemiacetal moiety and a hydroxyl group located at C-11 and C-2, respectively. Major difference between these two compounds was that the resonances of double bond between C-7 and C-8 in **4** were missing, instead an oxymethine at *δ*_C_ 59.6 and an oxygen-bearing quaternary carbon at *δ*_C_ 65.2 appeared in **2**. The HMBC correlations from H_2_-12 (*δ*_H_ 3.75 and 4.00) to C-7 (*δ*_C_ 59.6) and C-8 (*δ*_C_ 65.2), and from H-7 (*δ*_H_ 3.38) to C-6 (*δ*_C_ 23.7) in **2**, suggested the presence of an epoxide group between C-7 and C-8 in **2**. The ROESY spectrum revealed that the relative configuration of **2** was similar to that of **4**, and the ROESY correlation of H-7 (*δ*_H_ 3.38) with H-12 (*δ*_H_ 3.75) indicated that H-7 and H-12 were at *β*-position, and thus the epoxide ring occupied *α*-position (Fig. 2). Therefore, the structure of **2** was concluded as 2*α*-hydroxy-7*α*,8*α*-epoxy-isodrimeninol (Fig. 1).

Compound **3** exhibited an [M + Na]^+^ ion peak at *m/z* 273.1462 in the HR-ESI-MS, corresponding to the molecular formula C_15_H_22_O_3_ with 5° of unsaturation, suggesting that **3** was also a sesquiterpenoid. Comparing its 1D NMR spectra data (Table 1) with those of dendocarbin B [17] indicated that **3** had the similar drimane skeleton with an ester carbonyl group located at C-11 and a double bond between C-7 and C-8, which were further confirmed by analyzing the HMBC and HSQC spectra of **3**. The oxymethine at *δ*_C_ 75.5 in **3** obviously appeared at lower field than the one in dendocarbin B (*δ*_C_ 63.6) [17], indicating that the position of the hydroxyl group shifted in **3**. In the HMBC spectrum of **3**, the correlations from Me-13 (*δ*_H_ 0.93) and Me-14 (*δ*_H_ 0.96) to the oxymethine carbon at *δ*_C_ 75.5 indicated that the hydroxyl group was assignable to C-3. The coupling pattern of H-3 (*δ*_H_ 3.44, br s) indicated an *α*-oriented configuration of 3-OH [19] (Fig. 2), which was supported by the ROESY cross-peak between H-3 and Me-13, and by the upfield shifted C-14 due to a *γ*-gauch effect from 3*α*-OH. Thus, the structure of **3** was established as 11-dehydro-3*α*-hydroxyisodrimeninol (Fig. 1).

Compound **5** was obtained as colorless oil and has a molecular formula of C_12_H_14_O_6_ as determined from HR-ESI-MS (*m/z* 249.1105 [M + Na]^+^), with 6° of unsaturation. In the ^1^H NMR spectrum (Table 2), a secondary methyl resonance at *δ*_H_ 1.57 (d, *J* = 6.7 Hz, 3H) and an oxymethine group at *δ*_H_ 5.46 (q, *J* = 6.7 Hz, 1H) were evident, indicating the existence of an AB_3_ system in **5**. A singlet at *δ*_H_ 5.17 (2H) and a pair of AB doublets at *δ*_H_ 4.35 (d, *J* = 11.3 Hz, 1H) and 4.10 (d, *J* = 11.3 Hz, 1H) were clearly shown, revealing the presence of two oxygenated methylene groups. In the low-field region, two *ortho*-coupled aromatic doublets at *δ*_H_ 7.65 (d, *J* = 7.6 Hz) and 8.19 (d, *J* = 7.6 Hz) were observed, indicative of a 1,2,3,4-tetra-substituted phenyl ring. The ^13^C NMR and DEPT spectra (Table 2) of **5** displayed the signals of one methyl group at *δ*_C_ 15.3, two oxygenated methylene groups at *δ*_C_ 58.7 and 67.3, two olefinic methine groups at *δ*_C_ 116.6 and 134.6, and six quaternary carbons (including one ester carbonyl group at *δ*_C_ 169.7; four olefinic carbons at *δ*_C_ 107.5, 159.0, 142.0, and 131.0; and an oxygen-bearing carbon at *δ*_C_ 72.6). These NMR data suggested the presence of an isochromenone derivative similar to gamahorin [20], which was isolated from the fungus *Epichloe typhina*. The HMBC correlations from H-5 (*δ*_H_ 7.65) to the oxygen-bearing quaternary carbon (*δ*_C_ 72.6), and from the AB doublets at *δ*_H_ 4.35 and 4.10 to C-3 (*δ*_C_ 79.2), C-4a (*δ*_C_ 142.0), and the oxygen-bearing quaternary carbon (*δ*_C_ 72.6), indicated the presence of hydroxyl groups at C-4 and C-10. In the ROESY spectrum, the correlations of H_2_-10 (*δ*_H_ 4.35 and 4.10) with H-3 (*δ*_H_ 5.46) indicated *β*-orientation of H_2_-10 and H-3 (Fig. 2). Therefore, the structure of **5** was determined as 4,10-dihydroxy-gamahorin (Fig. 1).

**Table 2:** ^1^H and ^13^C NMR data of **5** (in pyridine-*d*_5_, at 600 and 150 MHz, resp.) [*δ* in ppm, *J* in Hz]

Position	*δ*_H_	*δ*_C_	Position	*δ*_H_	*δ*_C_
1	–	169.7 s	8	–	159.0 s
3	5.46 q (6.7)	79.2 d	8a	–	107.5 s
4	–	72.6 s	9	1.57 d (6.7, 3H)	15.3 q
4a	–	142.0 s	10	4.35 d (11.3)	67.3 t
5	7.65 d (7.6)	116.6 d		4.10 d (11.3)	
6	8.19 d (7.6)	134.6 d	11	5.17 s (2H)	58.7 t
7	–	131.0 s			

A known drimane sesquiterpenoid was also isolated and identified as 2*α*-hydroxyisodrimeninol (**4**) [17, 18], by comparing its NMR data with those previously reported in the literature.

Dramine sesquiterpenoids have been shown to possess extensive biologically activities, such as antifeedant, anti-inflammatory, cytotoxic, antioxidant and *α*-amylase inhibitory activities [21–23]. Antibacterial activity of **1**–**5** against *Staphylococcus aureus*, *Bacillus subtilis* and *Micrococcus luteus* were evaluated in this study. Compound **3** showed weak inhibitory effect on *B. subtilis* with IC_50_ value of 280.27 μM. However, none of these compounds showed obvious activity against *S. aureus* and *M. luteus*. Despite, our results indicated that *Pestalotiopsis* sp. M-23 as a prolific resource of characteristic dramine sesquiterpenoids, is an interesting endophyte worthy of further in-depth investigation.

## Experiments

### General Experimental Procedures

Column chromatographies were performed on 200–300 mesh silica gel (Qingdao Marine Chemical Factory, P. R. China), Sephadex LH-20 (25–100 μm, GE Healthcare), and ODS (75 μm, YMC gel). Optical rotations were measured on a Horiba-SEAP-300 spectropolarimeter. UV spectral data were obtained on a Shimadzu-210A double-beam spectrophotometer. IR spectra were recorded on a Bruker-Tensor-27 spectrometer with KBr pellets. NMR experiments were carried out on either a Bruker AM-400 or an Avance-600 spectrometer with TMS as internal standard. ESI-MS and HR-ESI-MS were recorded on an API-QSTAR-TOF. EI-MS and HR-EI-MS were recorded on an Autospec Premier P776 mass spectrometer. Semi-preparative HPLC analyses were performed on an Agilent 1200 series instrument equipped with a quaternary pump, a vacuum degasser, an autosampler, a thermostated column compartment and a diode array detector.

### Fungal Strain Isolation and Identification

Healthy, asymptomatic leaves of *L. canum* were harvested at Kunming Botanical Garden, P. R. China, in March 2014, and disinfected with 75 % EtOH for 10 s, and then rinsed with sterile distilled water for 5 times. The surface disinfected leaves were aseptically cut into 2 cm × 2 cm pieces, and cultivated in potato dextrose agar (PDA) plates containing 30 μg/mL streptomycin to inhibit the bacterial growth at 28 °C. A pure strain coded as M-23 was obtained by transferring monosporic isolates to fresh PDA gradually. M-23 was incubated in potato dextrose broth (PDB) at 28 °C in 100 mL Erlenmeyer flask for 3 days and shaking at 220 rpm.

Genomic DNA of M-23 was extracted from fungal mycelia (20 mg) grown in PBD using fungal DNA isolation mini kit (Sangon, Shanghai, China). The PCR reaction was performed using isolated genomic DNA as template, ITS5/ITS4 as primer pairs. The amplified DNA fragment was purified and sequenced using the same primer pairs by BGI Inc. The obtained DNA sequence data were searched for similar sequences in GenBank.

M-23 strain, whose ITS sequence data were found to be identical to those of *Pestalotiopsis clavata* MFLUCC 12-0268 (NR120182), *P. neglecta* Q13DW (EF055210) and *P. lespedezae* SY16E (EF055205) using BLAST search in NCBI, was finally identified as *Pestalotiopsis* sp.. The obtained sequence data were submitted to and deposited at GenBank (Accession No. KT372852).

### Fermentation and Isolation

The *Pestalotiopsis* sp. M-23 was cultivated on autoclaved rice media (300 g) at room temperature for 40 days. Then the fermented materials were extracted 5 times with acetone (600 mL for each time) and concentrated to give a crude extract (44.7 g). The crude extract was chromatographed using silica gel column with CHCl_3_/acetone (from 10:0 to 0:10, v/v) to give seven fractions, Fr. A-G. Fr. C (2.9 g) was subjected to silica gel column chromatography using petroleum ether (PE)/acetone (8:1, v/v) as eluent to afford nine subfractions, Fr. C_1_–C_9_. Fr. C_6_ (20 mg) was further chromatographed on a Sephadex LH-20 column eluting with CHCl_3_/MeOH (1:1, v/v) and purified by reversed-phase semi-preparative HPLC (80 % MeOH in H_2_O) to yield **3** (4 mg). Fr. E (1.1 g) was subjected to ODS column chromatography eluting with MeOH/H_2_O (from 1:1 to 10:0) to afford seven subfractions, E_1_–E_7_. Fr. E_2_ (30 mg) was purified by reversed-phase semi-preparative HPLC (75 % MeOH in H_2_O) to yield **5** (5 mg). Fr. E_3_ (48 mg) was subjected to silica gel column chromatography using PE/acetone (2:1, v/v) as eluent to yield **1** (6 mg). In the same way, Fr. E_4_ (21 mg) and Fr. E_6_ (17 mg) were subjected to silica gel column chromatographies to yield **4** (PE/acetone 3:1, 3 mg) and **2** (PE/acetone 5:2, 9 mg), respectively.

### 2*α*,8*α*-Dihydroxy-6,7-en-isodrimeninol (**1**)

Colorless oil; [*α*]_D_^26.1^ −101.1 (*c* 0.3, MeOH); UV (MeOH) *λ*_max_ (log *ε*) 201 (3.57) nm; IR(KBr) *ν*_max_ 3425, 2955, 2925, 1630, 1580, 1461, 1033 cm^−1^; ^1^H and ^13^C NMR: Table 1. ESI-MS *m/z* 291 [M + Na]^+^; HR-ESI-MS: 291.1570 (calcd for 291.1567).

### 2*α*-Hydroxy-7*α*,8*α*-epoxy-isodrimeninol (**2**)

Colorless oil; [*α*]_D_^25.3^ −27.6 (*c* 0.3, MeOH); UV (MeOH) *λ*_max_ (log *ε*) 201 (3.02), 215 (2.94) nm; IR(KBr) *ν*_max_ 3430, 2958, 2816; ^1^H and ^13^C NMR see Table 1; HR-EI-MS *m/z* 268.1677 [M]^+^ (calcd for 268.1675).

### 11-Dehydro-3*α*-hydroxyisodrimeninol (**3**)

Colorless oil; [*α*]_D_^25.8^ −14.7 (*c* 0.2, MeOH); UV (MeOH) *λ*_max_ (log *ε*) 213 (3.61) nm; IR(KBr) *ν*_max_ 3439, 2957, 2930, 2874, 1758, 1688, 1385, 1014; ^1^H and ^13^C NMR see Table 1; ESI-MS *m/z* 273 [M + Na]^+^; HR-ESI-MS 273.1462 (calcd for 273.1461).

### 4,10-Dihydroxy-gamahorin (**5**)

Colorless oil; [*α*]_D_^25.9^ +16.0 (*c* 0.2, MeOH); UV (MeOH) *λ*_max_ (log *ε*) 211 (4.39), 244 (3.70), 318 (3.57) nm; IR(KBr) *ν*_max_ 3426, 2927, 1667, 1621, 1430, 1384, 1122, 1058; ^1^H and ^13^C NMR see Table 2. ESIMS 254 [M + Na]^+^; HR-ESI-MS 249.1105 (calcd for 249.1097).

### Antibacterial Tests

Antibacterial activity of **1**–**5** against *Staphylococcus aureus*, *Bacillus subtilis* and *Micrococcus luteus* were evaluated using broth dilution method [24] with modification. Briefly, test compound was dissolved in proper solvent to obtain the highest concentration of 10.24 mg/mL. Serial dilutions of mother solution were performed with final concentration ranging from 512, 256, 128, 64, 32, 16 and 0 μg/mL. Ampicillin was used as positive control. All assays were performed in triplicate. The results were expressed as the minimum concentration inhibiting 50 % of bacterial growth (IC_50_) [25]. The IC_50_ values were calculated after 24 h of growth at 37 °C.

## Electronic supplementary material

Below is the link to the electronic supplementary material.

Supplementary material 1 (DOCX 2384 kb)
