# Supplementary material for: Drimane Sesquiterpenoids and Isochromone Derivative from the Endophytic Fungus Pestalotiopsis sp. M-23
Source: Nat Prod Bioprospect. 2016 Apr 2;6(3):155–60. doi: 10.1007/s13659-016-0094-6 (PMC5385658; doi:10.1007/s13659-016-0094-6)

**Supplementary data**

**Drimane Sesquiterpenoids and Isochromone Derivative from the Endophytic Fungus *Pestalotiopsis* sp. M-23**

**Ce Kuang^a,b^**, **Shu-Xi Jing ^a^**, **Yan Liu ^a^**, **Shi-Hong Luo**^a,^*, **Sheng-Hong Li**^a,^*

^a^ State Key Laboratory of Phytochemistry and Plant Resources in West China, Kunming Institute of Botany, Chinese Academy of Sciences(CAS), Kunming 650201, PR China

^b^ University of Chinese Academy of Sciences, Beijing 100049, PR China

**Corresponding Authors**

* Sheng-Hong Li & Shi-Hong Luo

*E-mail*: [shli@mail.kib.ac.cn & luoshihong@mail.kib.ac.cn](mailto:shli@mail.kib.ac.cn%20&%20luoshihong@mail.kib.ac.cn)

Tel/Fax: +86-0871-65223035

Contents

**[Figure S1.](#_Toc445825690) ^[1](#_Toc445825690)^[H NMR spectrum of compound 1 (CD](#_Toc445825690)_[3](#_Toc445825690)_[OD, 400 MHz) 3](#_Toc445825690)**

**[Figure S2.](#_Toc445825691) ^[13](#_Toc445825691)^[C NMR and DEPT spectra of compound 1 (CD](#_Toc445825691)_[3](#_Toc445825691)_[OD, 150 MHz) 3](#_Toc445825691)**

[**Figure S3. HSQC spectrum of compound 1 (CD_3_OD) 4**](#_Toc445825692)

[**Figure S4. HMBC spectrum of compound 1 (CD_3_OD) 4**](#_Toc445825693)

[**Figure S5. ^1^H-^1^HCOSY spectrum of compound 1 (CD_3_OD) 5**](#_Toc445825694)

[**Figure S6. ROESY spectrum of compound 1 (CD_3_OD) 5**](#_Toc445825695)

[**Figure S7 HRESIMS spectrum of compound 1 6**](#_Toc445825696)

[**Figure S8. ^1^H NMR spectrum of compound 2 (CD_3_OD, 400 MHz) 6**](#_Toc445825697)

[**Figure S9. ^13^C NMR and DEPT spectra of compound 2 (CD_3_OD, 150 MHz) 7**](#_Toc445825698)

[**Figure S10. HSQC spectrum of compound 2 (CD_3_OD) 7**](#_Toc445825699)

[**Figure S11. HMBC spectrum of compound 2 (CD_3_OD) 8**](#_Toc445825700)

[**Figure S12. ^1^H-^1^H COSY spectrum of compound 2 (CD_3_OD) 8**](#_Toc445825701)

[**Figure S13. ROESY spectrum of compound 2 (CD_3_OD) 9**](#_Toc445825702)

[**Figure S14. HREIMS spectrum of compound 2 9**](#_Toc445825703)

[**Figure S15. ^1^H NMR spectrum of compound 3 (acetone-*d*_6_, 400 MHz) 10**](#_Toc445825704)

[**Figure S16. ^13^C NMR and DEPT spectra of compound 3 (acetone-*d*_6_, 100 MHz) 10**](#_Toc445825705)

[**Figure S17. HSQC spectrum of compound 3(acetone-*d*_6_) 11**](#_Toc445825706)

[**Figure S18. HMBC spectrum of compound 3 (acetone-*d*_6_) 11**](#_Toc445825707)

[**Figure S19. ^1^H-^1^H COSY spectrum of compound 3 (acetone-*d*_6_) 12**](#_Toc445825708)

[**Figure S20. ROESY spectrum of compound 3 (acetone-*d*_6_) 12**](#_Toc445825709)

[**Figure S21. HRESIMS spectrum of compound 3 13**](#_Toc445825710)

[**Figure S22. ^1^H NMR spectrum of compound 4 (acetone-*d*_6_, 400 MHz) 13**](#_Toc445825711)

[**Figure S23. ^13^C NMR and DEPT spectra of compound 4 (acetone-*d*_6_, 100 MHz) 14**](#_Toc445825712)

[**Figure S24. ^1^H NMR spectrum of compound 5 (pyridine-*d*_5_, 400 MHz) 14**](#_Toc445825713)

[**Figure S25. ^13^C NMR and DEPT spectra of compound 5 (pyridine-*d*_5_, 150 MHz) 15**](#_Toc445825714)

[**Figure S26. HSQC spectrum of compound 5 (pyridine-*d*_5_) 15**](#_Toc445825715)

[**Figure S27. HMBC spectrum of compound 5 (pyridine-*d*_5_) 16**](#_Toc445825716)

[**Figure S28. ^1^H-^1^H COSY spectrum of compound 5 (pyridine-*d*_5_) 16**](#_Toc445825717)

[**Figure S29. ROESY spectrum of compound 5 (pyridine-*d*_5_) 17**](#_Toc445825718)

[**Figure S30. HREIMS spectrum of compound 5 (pyridine-*d*_5_) 18**](#_Toc445825719)

Figure S1. ^1^H NMR spectrum of compound 1 (CD_3_OD, 400 MHz)


Figure S2. ^13^C NMR and DEPT spectra of compound 1 (CD_3_OD, 150 MHz)

# Figure S3. HSQC spectrum of compound 1 (CD_3_OD)

**

Figure S4. HMBC spectrum of compound 1 (CD_3_OD)

# Figure S5. ^1^H-^1^HCOSY spectrum of **compound 1 (CD_3_OD)**

# Figure S6. ROESY spectrum of compound 1 (CD_3_OD)

**

Figure S7 HRESIMS spectrum of compound 1


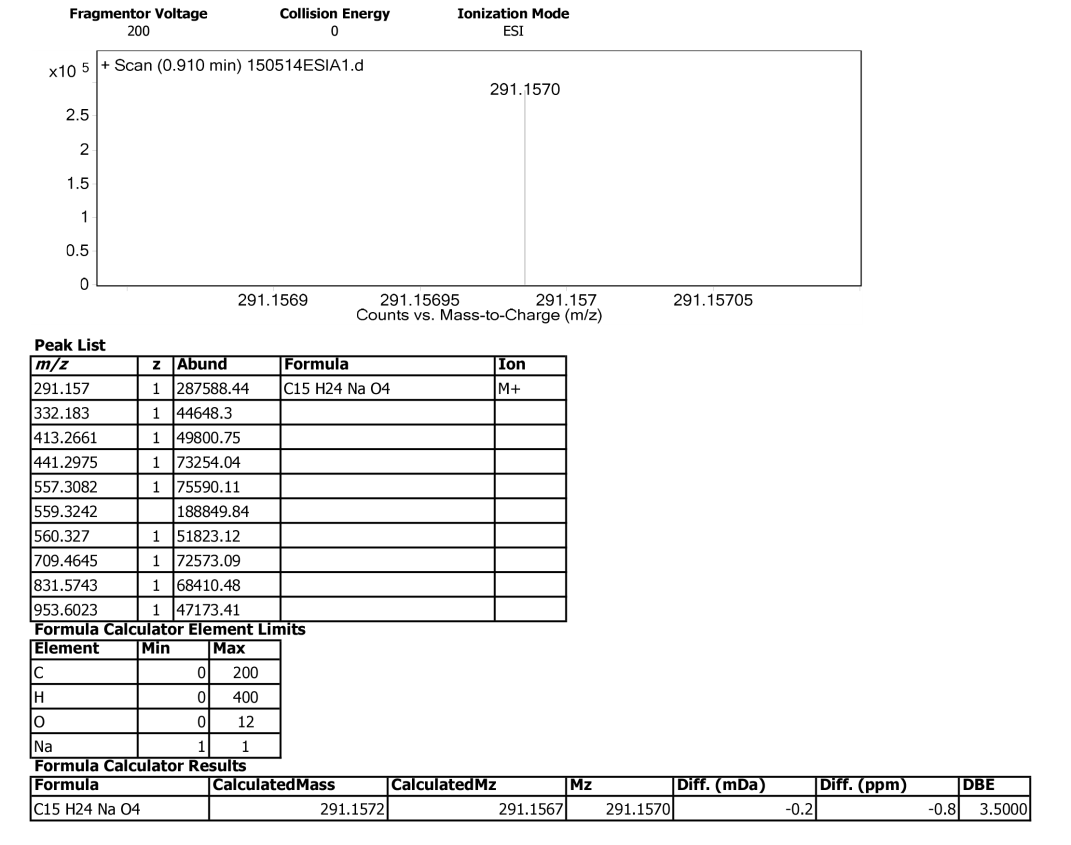

# Figure S8. ^1^H NMR spectrum of compound 2 (CD_3_OD, 400 MHz)

Figure S9. ^13^C NMR and DEPT spectra of compound 2 (CD_3_OD, 150 MHz)

# Figure S10. HSQC spectrum of **compound 2 (CD_3_OD)**

Figure S11. HMBC spectrum of compound 2 (CD_3_OD)

# Figure S12. ^1^H-^1^H COSY spectrum of compound 2 (CD_3_OD)

Figure S13. ROESY spectrum of compound 2 (CD_3_OD)

Figure S14. HREIMS spectrum of compound 2

**
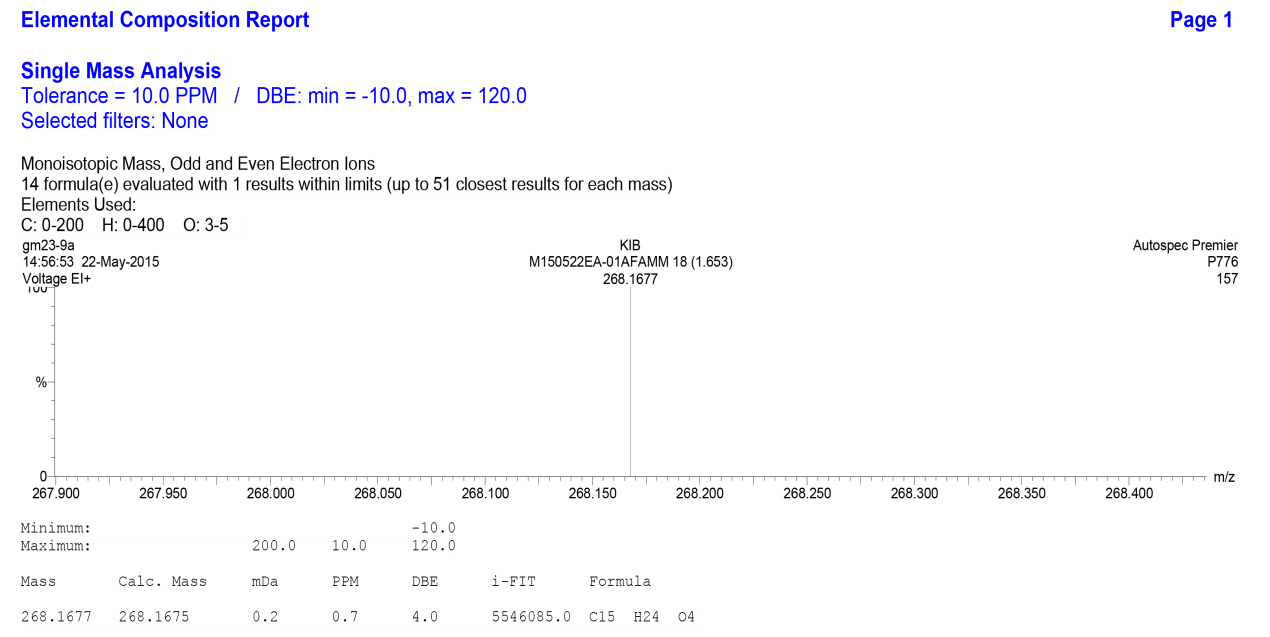
**

Figure S15. ^1^H NMR spectrum of compound 3 (acetone-*d*_6_, 400 MHz)

Figure S16. ^13^C NMR and DEPT spectra of compound 3 (acetone-*d*_6_, 100 MHz)

# Figure S17. HSQC spectrum of compound 3(acetone-*d*_6_)

# Figure S18. HMBC spectrum of compound 3 (acetone-*d*_6_)

Figure S19. ^1^H-^1^H COSY spectrum of compound 3 (acetone-*d*_6_)

# Figure S20. ROESY spectrum of compound 3 (acetone-*d*_6_)

__

# Figure S21. HRESIMS spectrum of compound 3

_
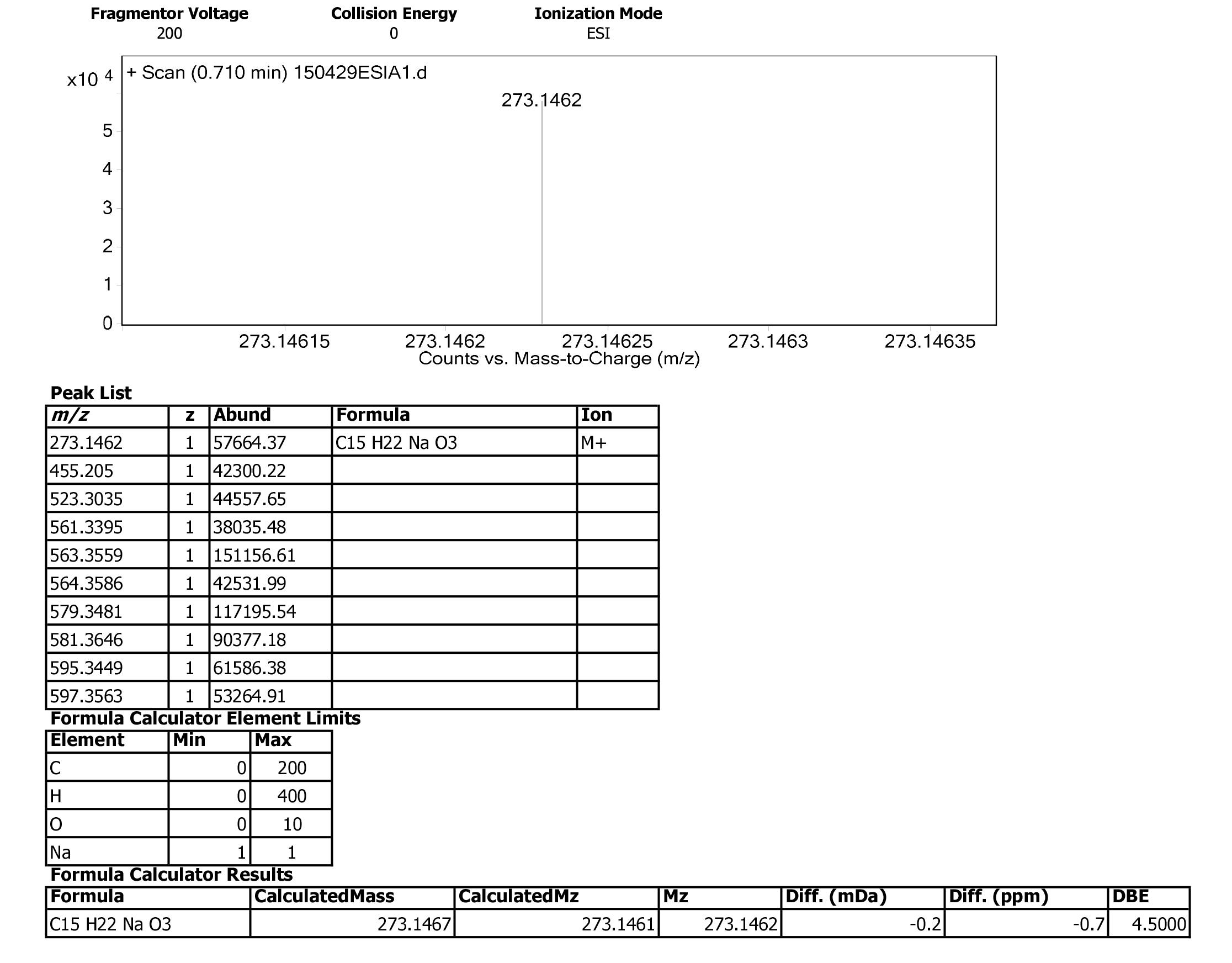
_

Figure S22. ^1^H NMR spectrum of compound 4 (acetone-*d*_6_, 400 MHz)

Figure S23. ^13^C NMR and DEPT spectra of compound 4 (acetone-*d*_6_, 100 MHz)

__

Figure S24. ^1^H NMR spectrum of compound 5 (pyridine-*d*_5_, 400 MHz)

Figure S25. ^13^C NMR and DEPT spectra of compound 5 (pyridine-*d*_5_, 150 MHz)

__

# Figure S26. HSQC spectrum of compound 5 (pyridine-*d*_5_)

Figure S27. HMBC spectrum of compound 5 (pyridine-*d*_5_)

Figure S28. ^1^H-^1^H COSY spectrum of compound 5 (pyridine-*d*_5_)

__

# Figure S29. ROESY spectrum of compound 5 (pyridine-*d*_5_)

# Figure S30. HREIMS spectrum of compound 5 (pyridine-*d*_5_)


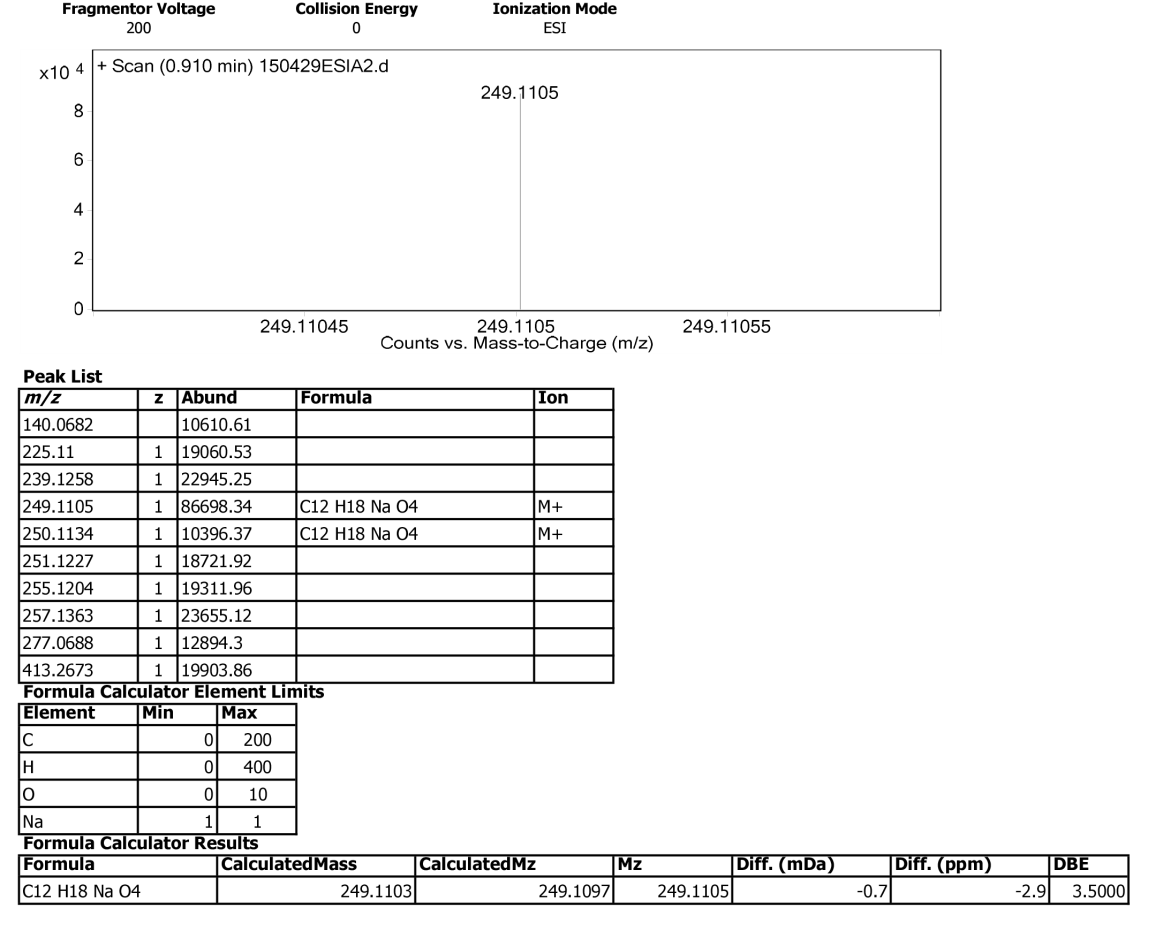

Supplement: Supplementary file 1 — Supplementary material 1 (DOCX 2384 kb) [file 13659_2016_94_MOESM1_ESM.docx]
